# Supplementary material for: Distinct Genomic Profiles Are Associated with Treatment Response and Survival in Ovarian Cancer
Source: Cancers (Basel). 2022 Mar 15;14(6):1511. doi: 10.3390/cancers14061511 (PMC8946149; doi:10.3390/cancers14061511)
Supplement: Supplementary file 1 [file cancers-14-01511-s001.zip › Supplementary Figures 1 to 13.pdf]

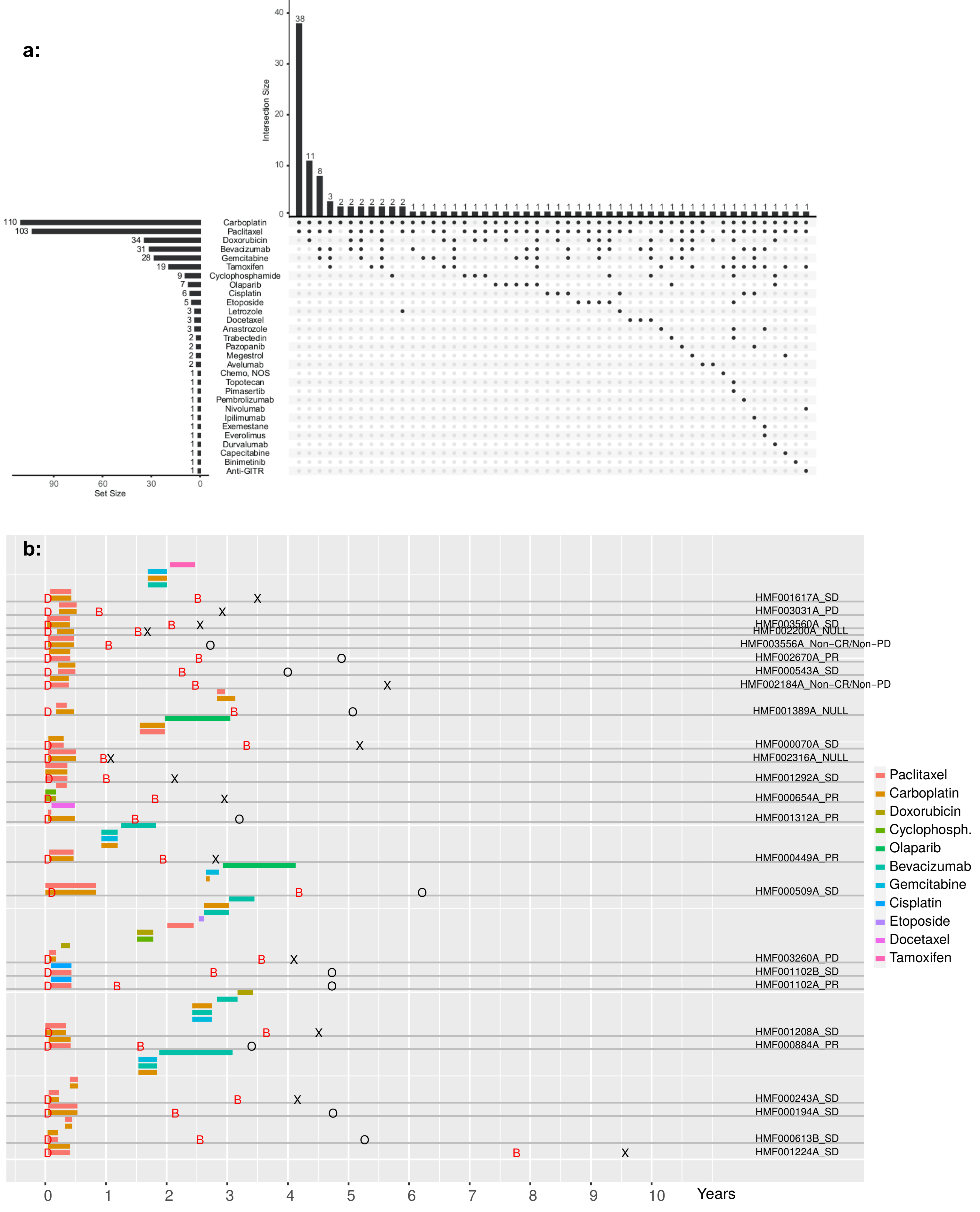

Figure S1. Treatments before biopsy and recurrence patterns. a: UpSet plot with pre-biopsy treatment history for samples that were obtained at recurrent disease (N=113). Horizontal bars (set size) indicate the number of patients that received a single drug. Vertical bars (intersection size) indicate the number of patients that received a combination of drugs. The majority of patients received a unique combination of systemic treatments.

b - e: Recurrence patterns of patients. A time line is depicted per patient. RECIST response (version 1.1) to subsequent platinum (combination) treatment added to each sample name. D=diagnosis, B=biopsy, X=death date, O=last follow-up. PD=progressive disease, SD=stable disease, PR=partial response, CR=complete response.

b: HGSC patients with one recurrence. **c,d,e**: see next page.

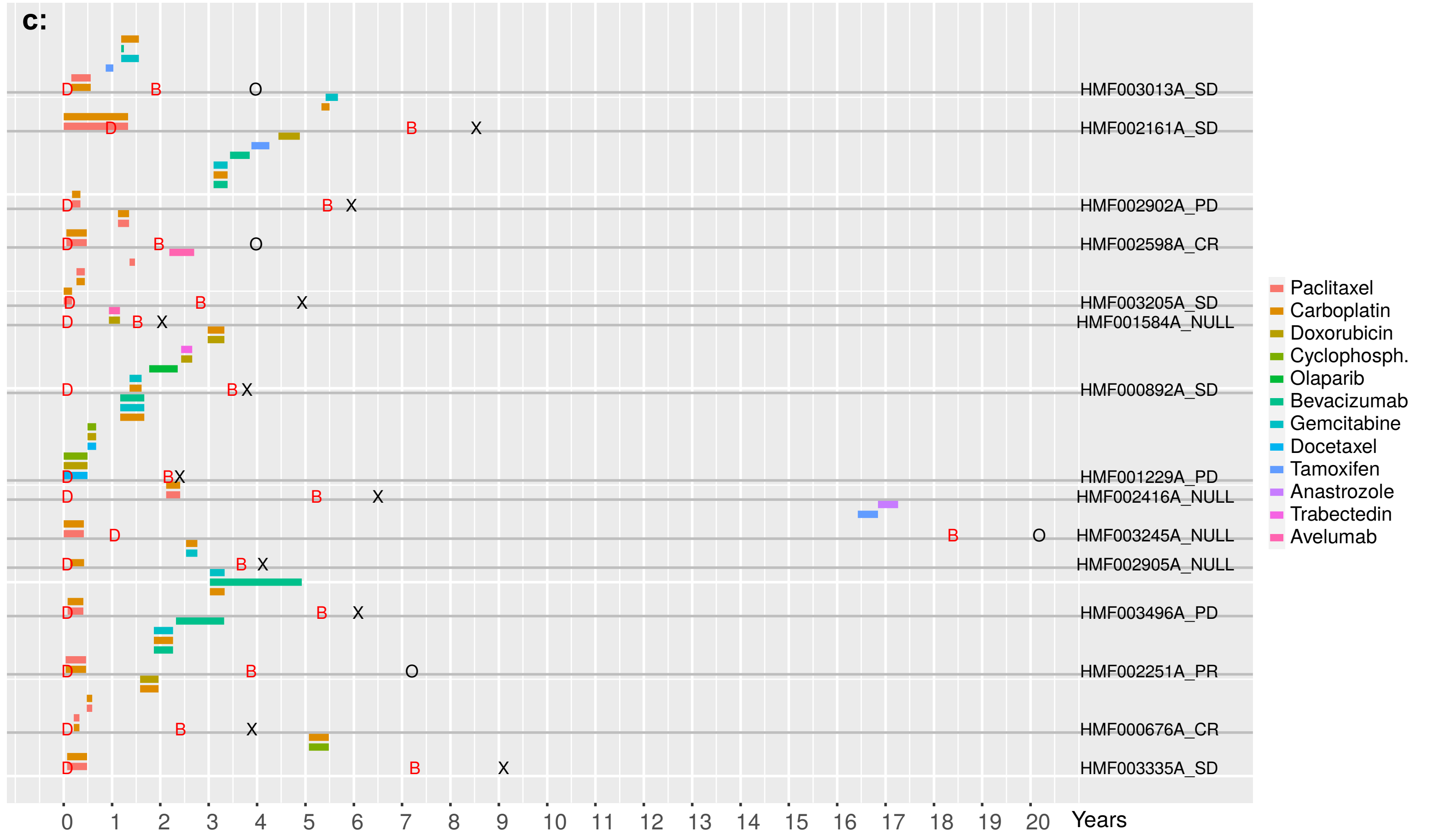

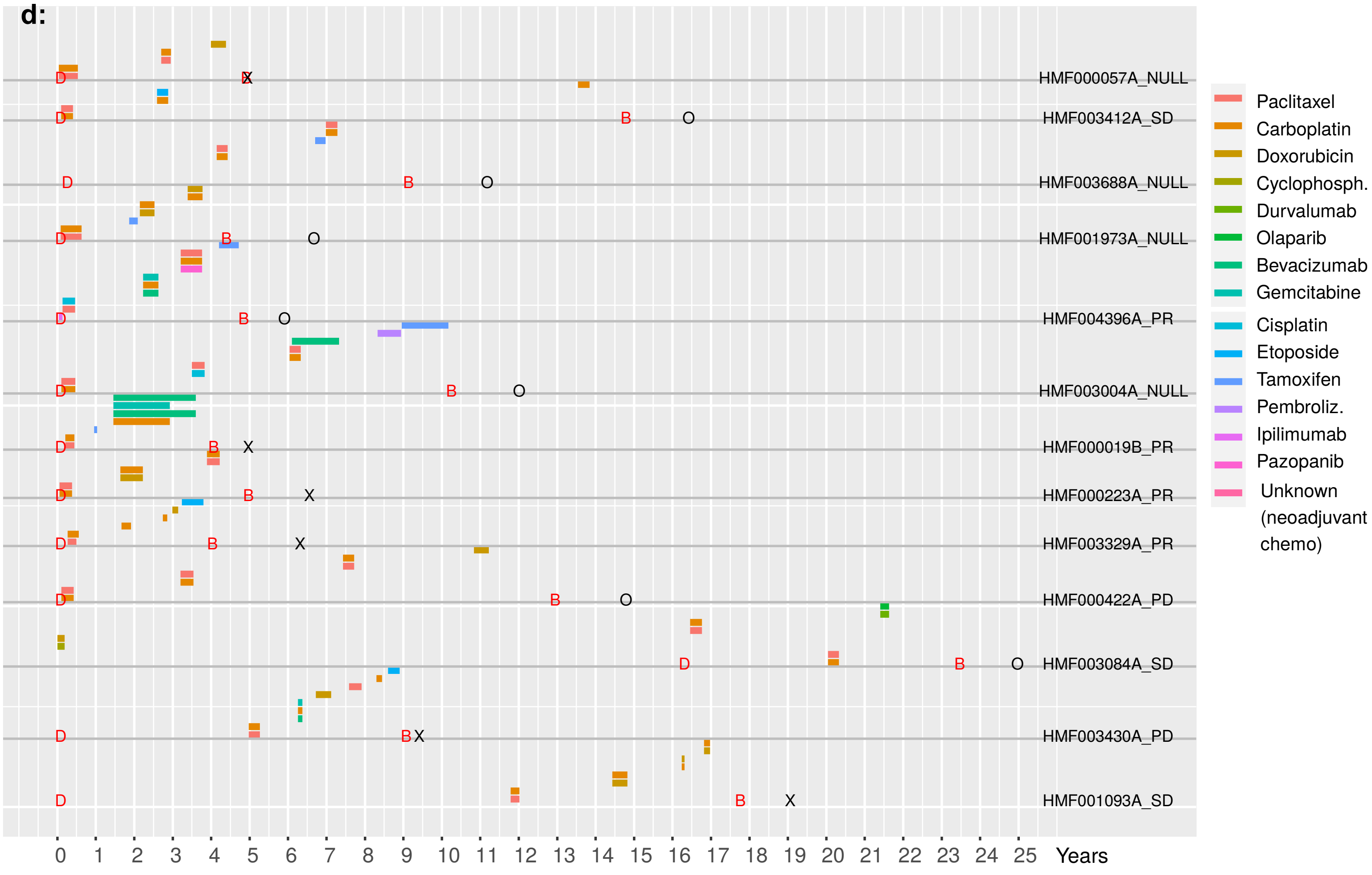

c: Treatment history and RECIST response of HGSC patients with two recurrences. **d:** Patients with more than two recurrences.

e:

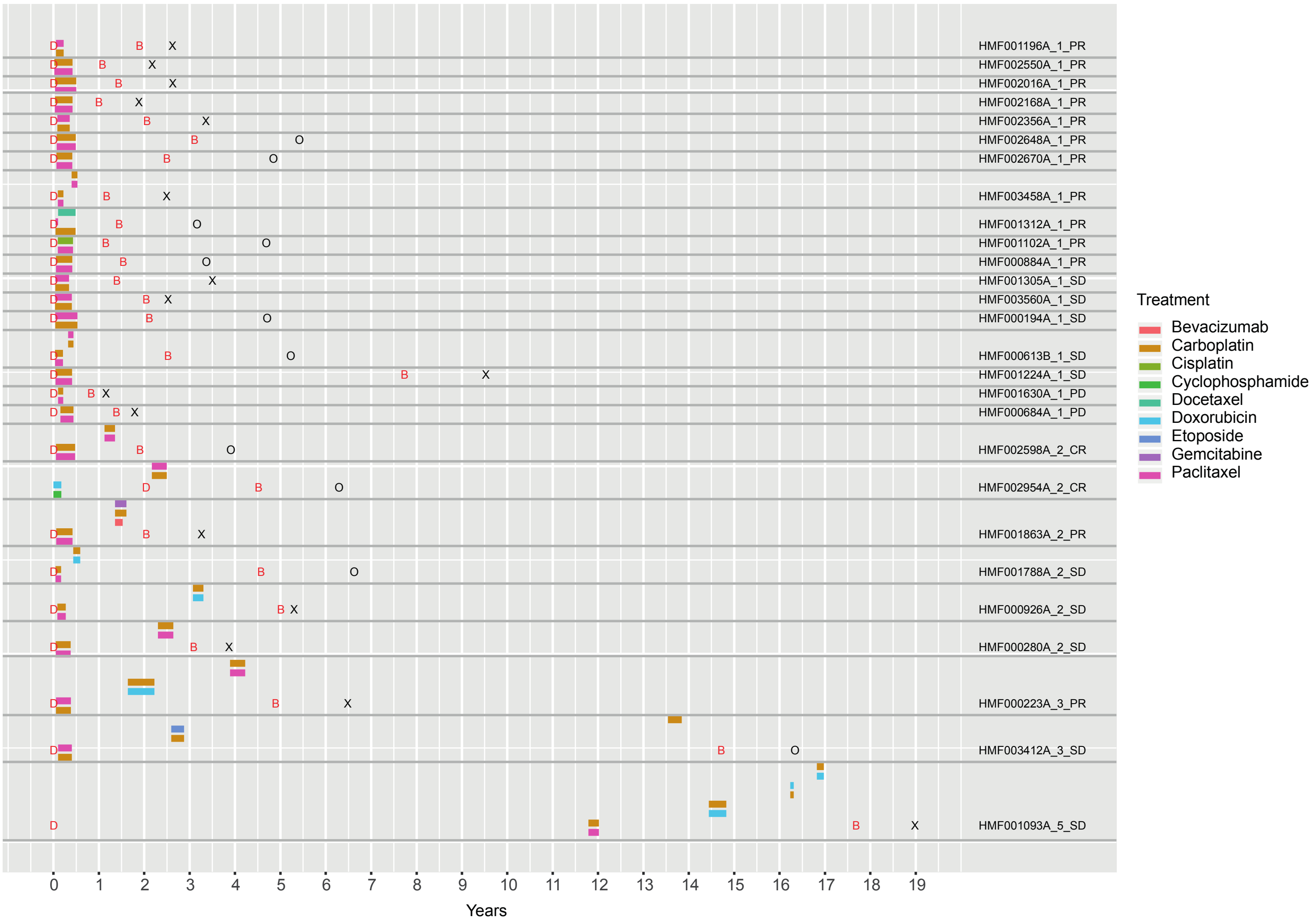

e: Patients included in the PFI analysis (Figure 2C) sorted on the number of recurrences (1-5) and the RECIST response.

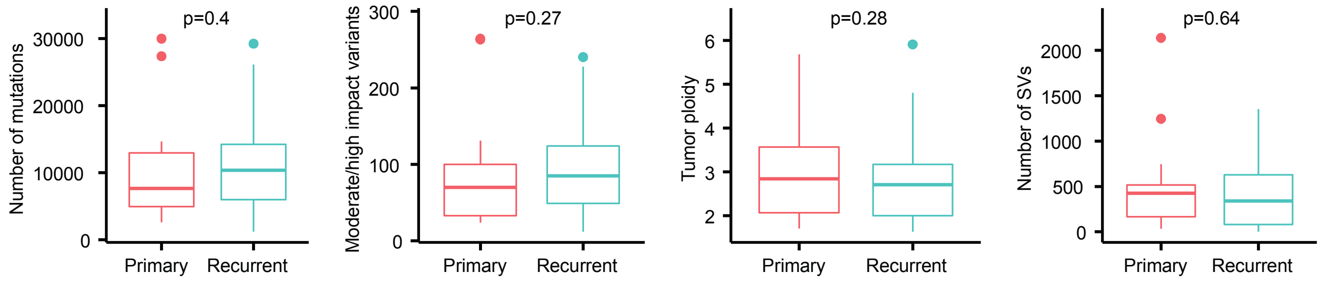

Figure S2. Primary-recurrence comparison. Primary (N=19) and recurrent samples (N=113) harbored a comparable number of mutations, number of moderate/high impact mutations, ploidy and number of SVs (p>0.05, Wilcoxon signed rank test).

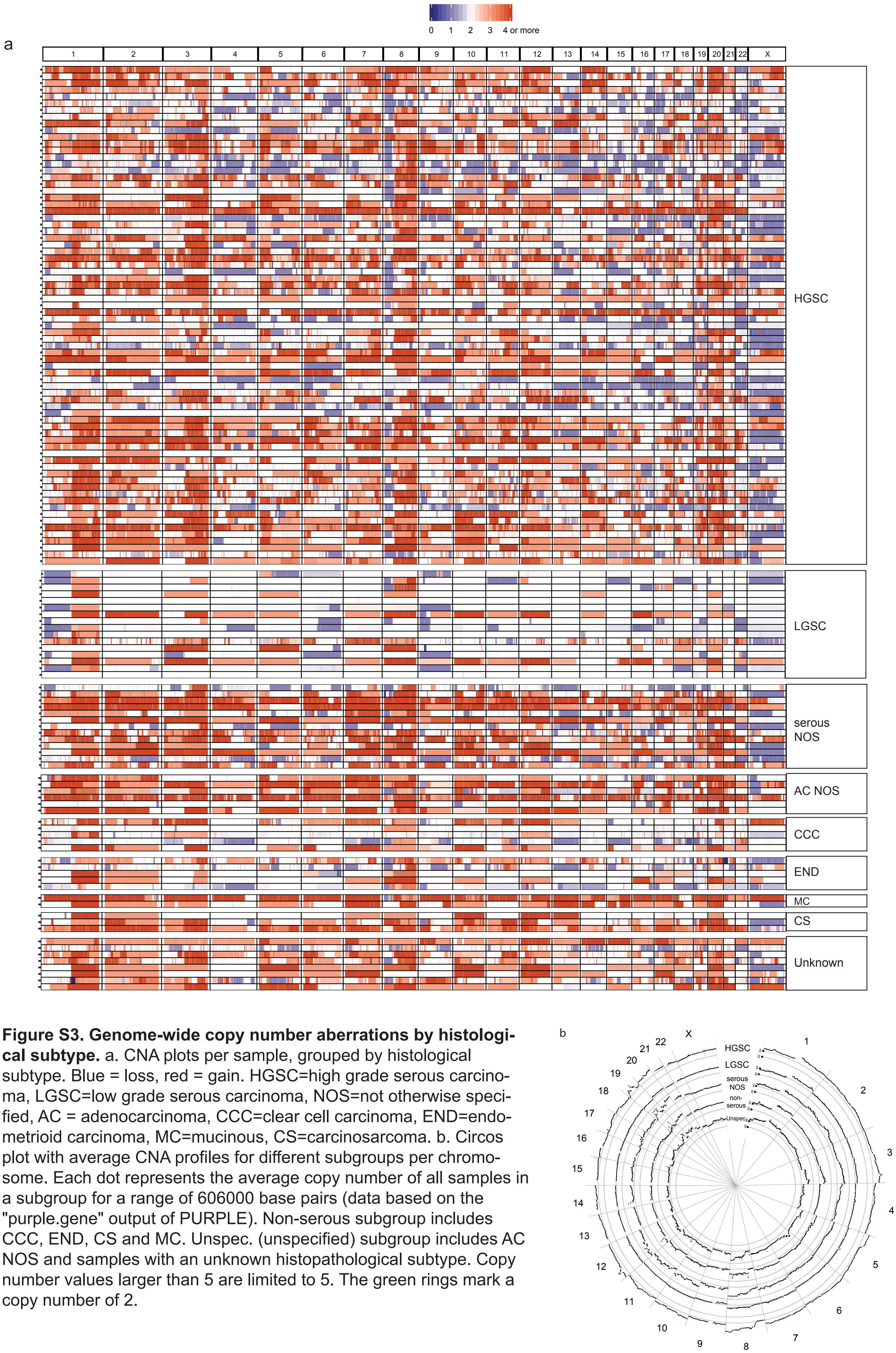

Figure S3. Genome-wide copy number aberrations by histological subtype. a. CNA plots per sample, grouped by histological subtype. Blue = loss, red = gain. HGSC=high grade serous carcinoma, LGSC=low grade serous carcinoma, NOS=not otherwise specified, AC = adenocarcinoma, CCC=clear cell carcinoma, END=endometrioid carcinoma, MC=mucinous, CS=carcinosarcoma. b. Circos plot with average CNA profiles for different subgroups per chromosome. Each dot represents the average copy number of all samples in a subgroup for a range of 606000 base pairs (data based on the "purple.gene" output of PURPLE). Non-serous subgroup includes CCC, END, CS and MC. Unspec. (unspecified) subgroup includes AC NOS and samples with an unknown histopathological subtype. Copy number values larger than 5 are limited to 5. The green rings mark a copy number of 2.

a:

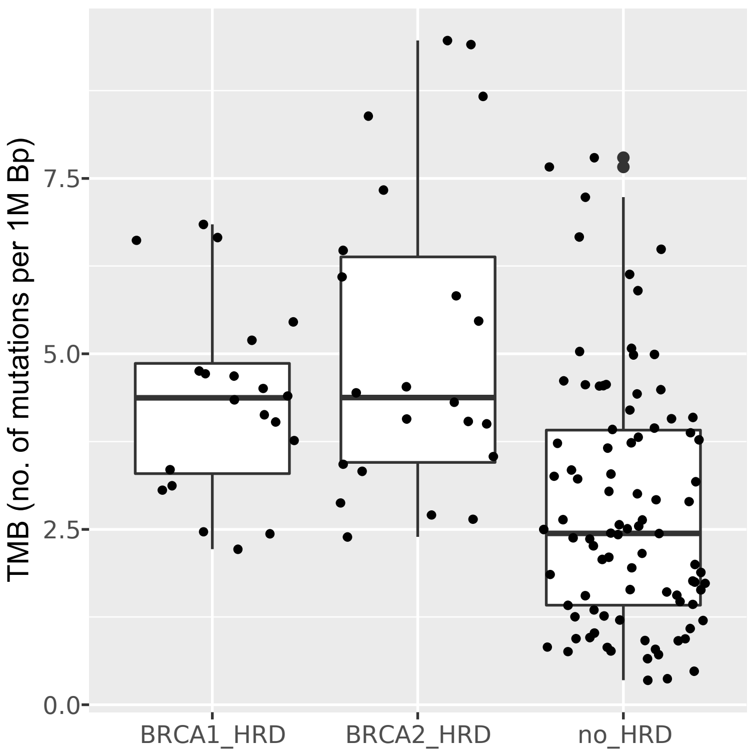

b:

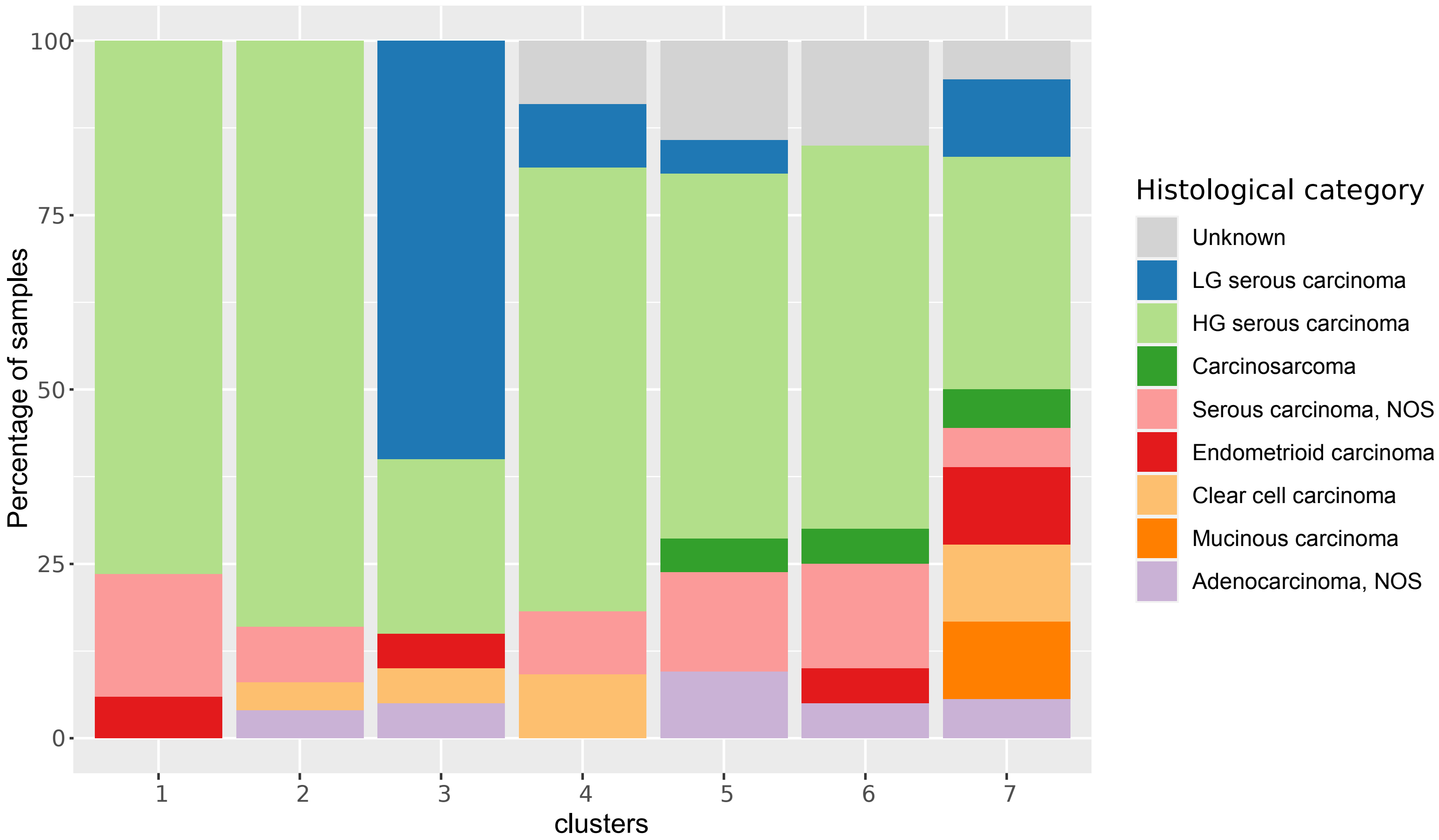

Figure S4: Tumor mutational burden of HRD samples and histological categories per cluster. a: TMB (number of mutations per 1M base pairs) for the samples with BRCA1-type HRD, BRCA2-type HRD and no HRD according to the CHORD classifier. **b:** Histological categories per cluster. The two clusters I and II (HRD clusters) have the largest fraction of HGSC samples.

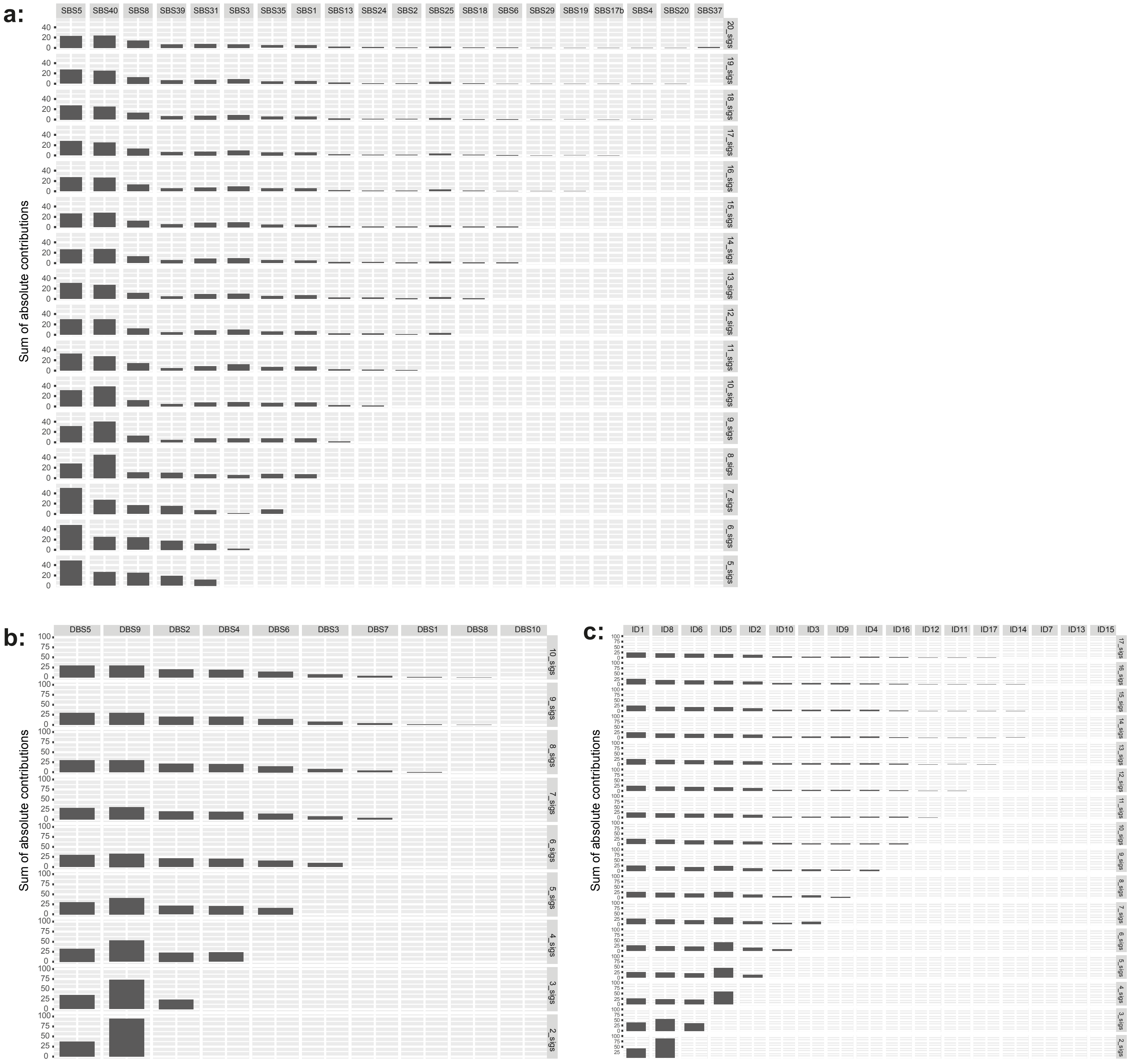

Figure S5 (a-c): Mutational signatures. Golden ratio analysis, sum of cohort contributions per signature for different numbers of COSMIC signatures SBS (**a:**), DBS (**b:**) and ID (**c:**). We identified major contributing profiles for eight SBS, five DBS and five ID signatures.

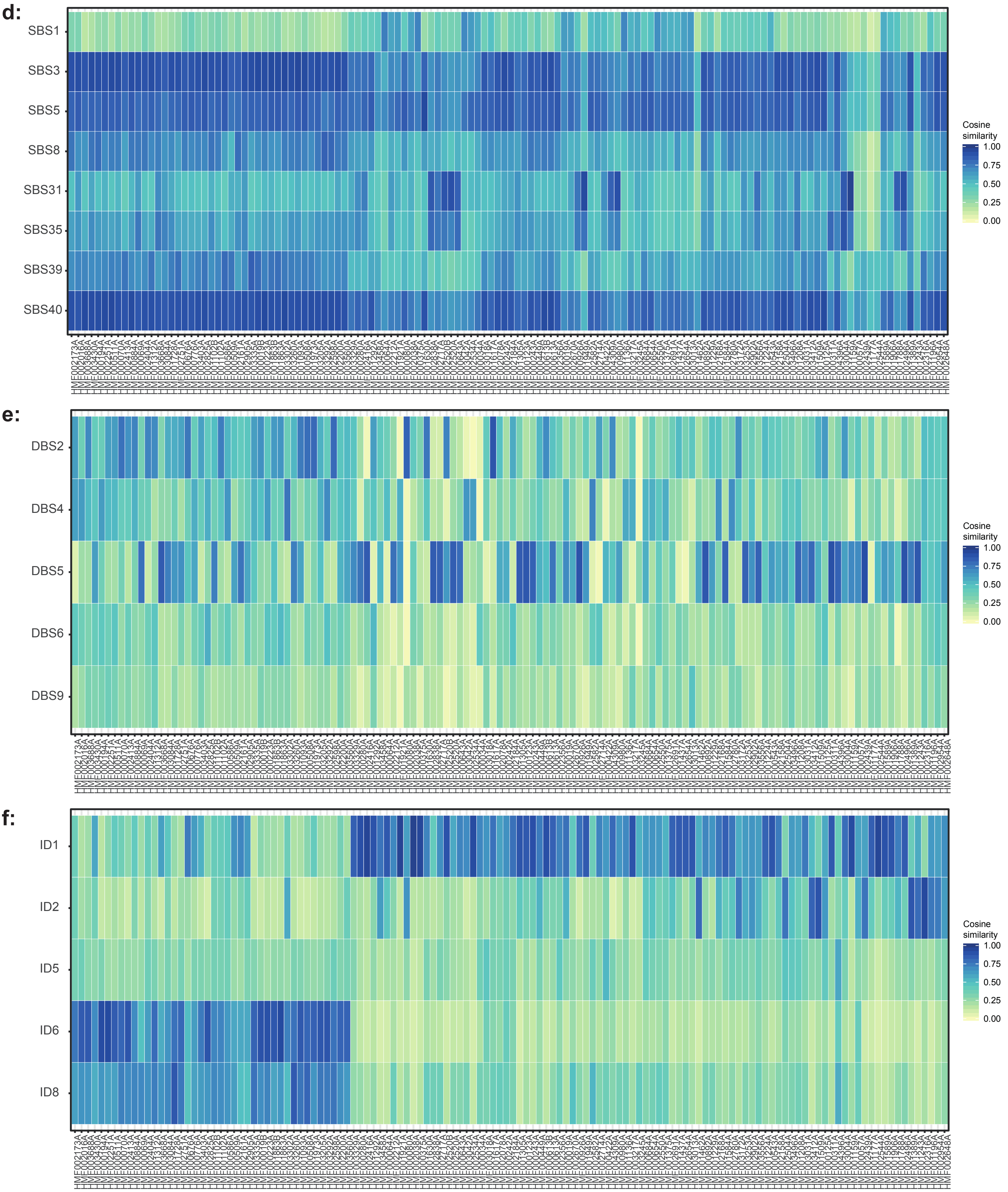

Figure S5 (d-f). Mutational signatures. The cosine similarity of the mutation context vector for each sample to the COSMIC signatures, for SBS (**d:**), DBS (**e:**) and ID (**f:**).

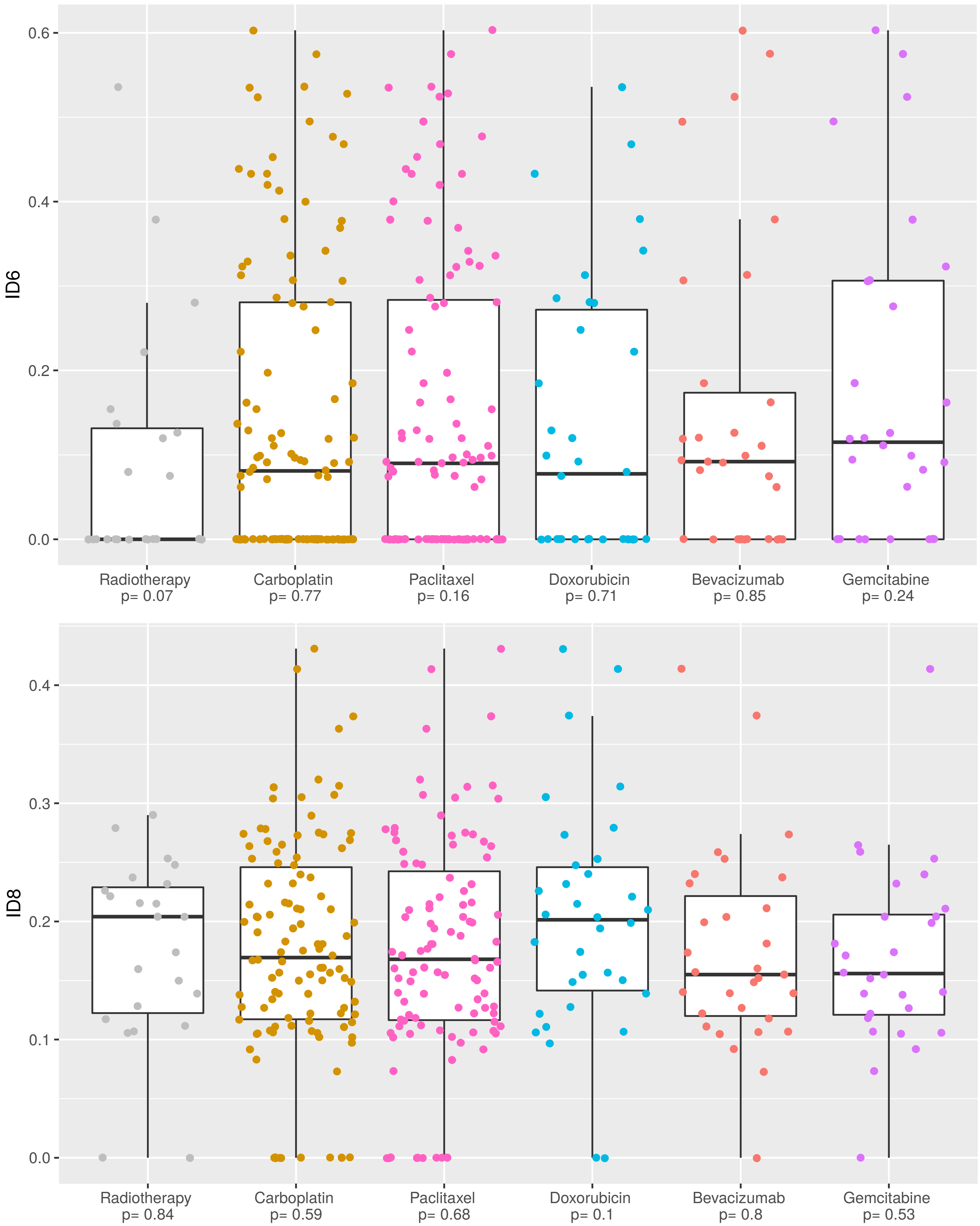

Figure S6: **Contribution of indel signatures ID6 and ID8 to the profiles for different treatments.** The p-values for each treatment were obtained from t-tests using all samples with that treatment vs. all other samples. As all patients got combination treatment, each patient is represented in multiple boxplots.

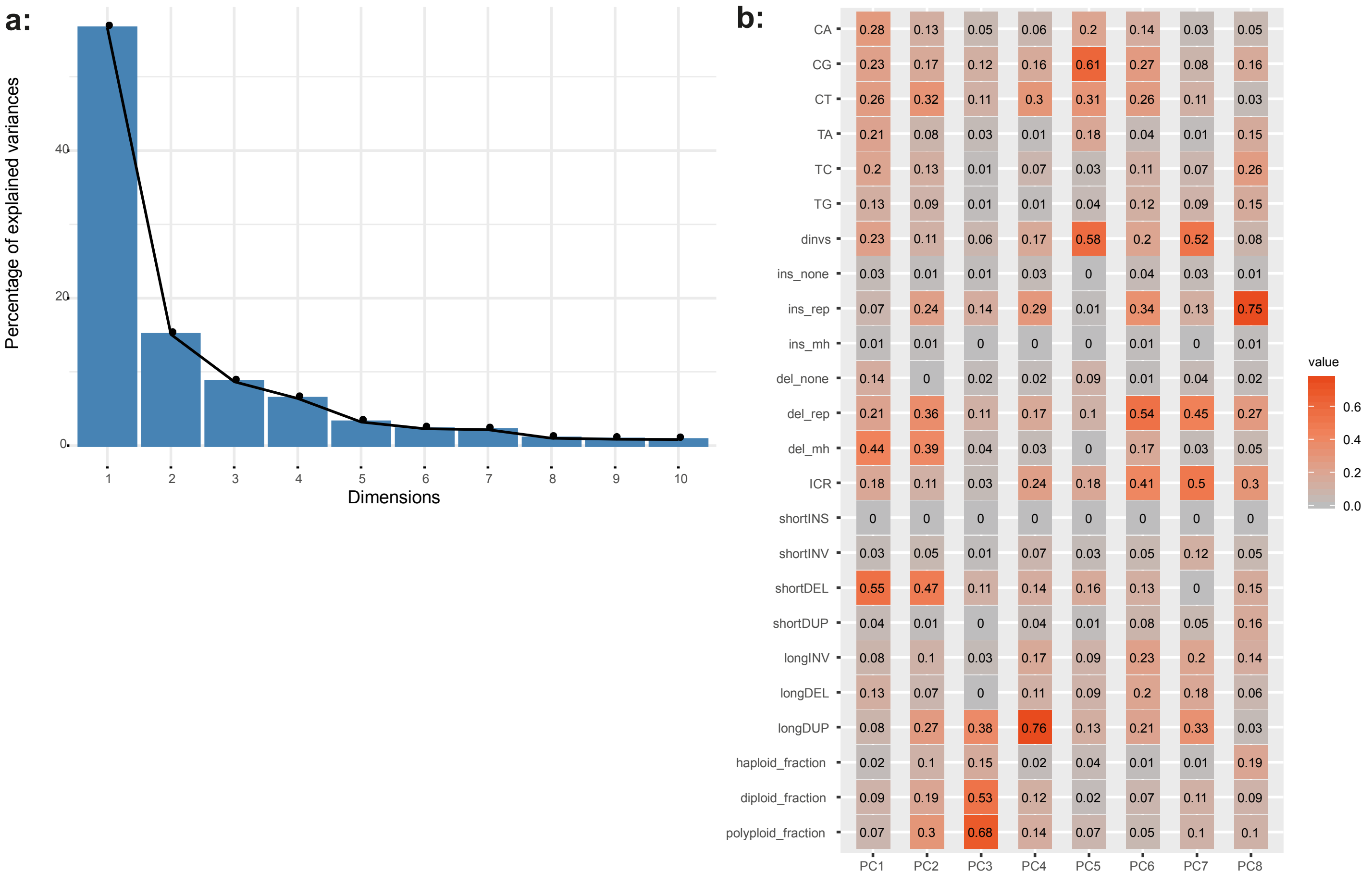

Figure S7: Principal components and feature contributions. a: PCA Dimensions, percentage of variance explained per dimension. **b:** PCA loadings per category. Short deletions and deletions with microhomology are the most important discriminating factors.

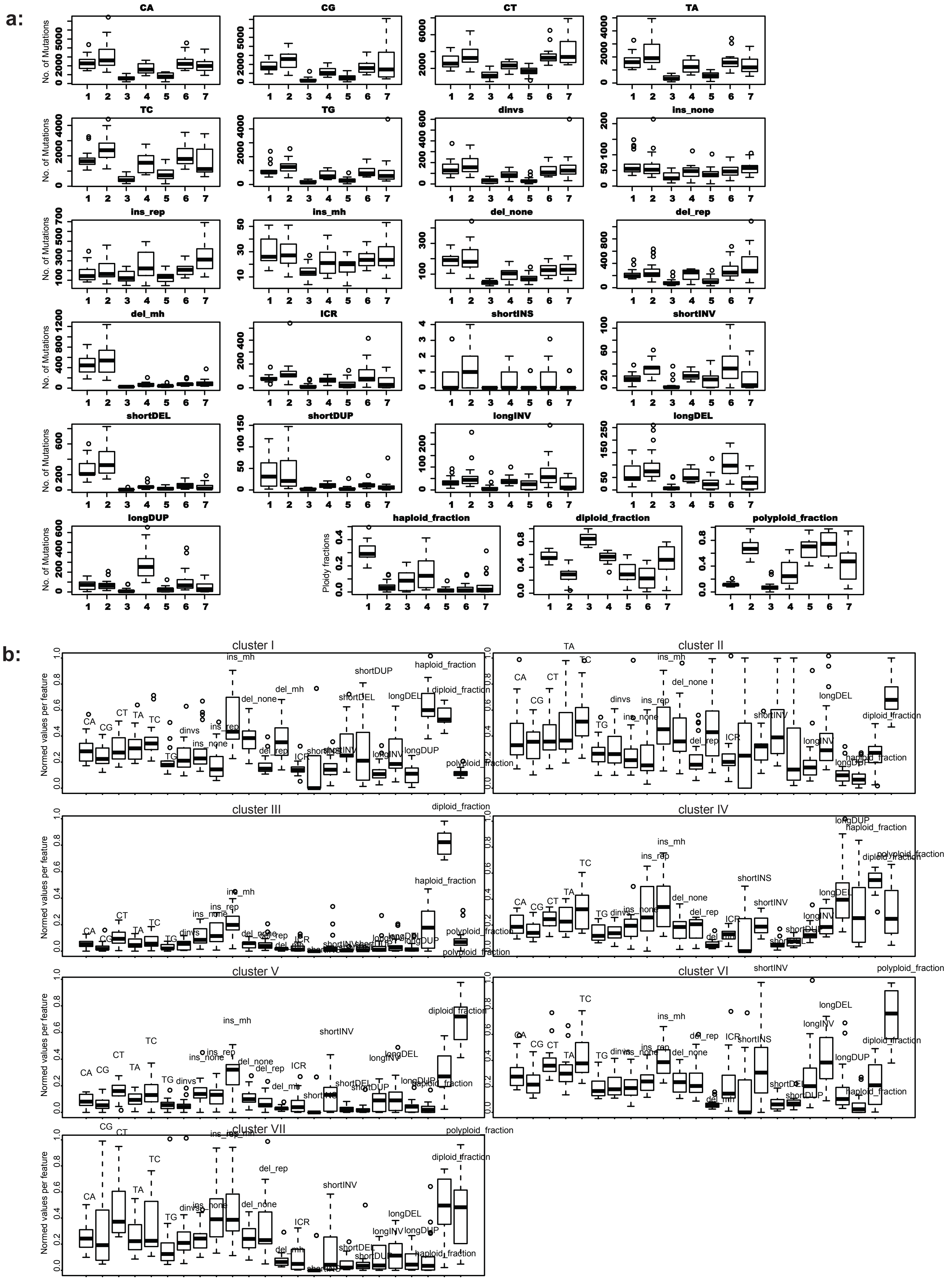

Figure S8: Distribution of features of the unsupervised hierarchical clustering; pvclust bootstrapping. a: Boxplots with all clusters per feature. **b:** Boxplots with all features per cluster. **c:** (next page) Bootstrapping of the unsupervised hierarchical clustering (pvclust method). Red squares highlight clusters with an approximated unbiased p-value (red numbers) of >94%, which indicates strong support of these clusters by the data.

c:

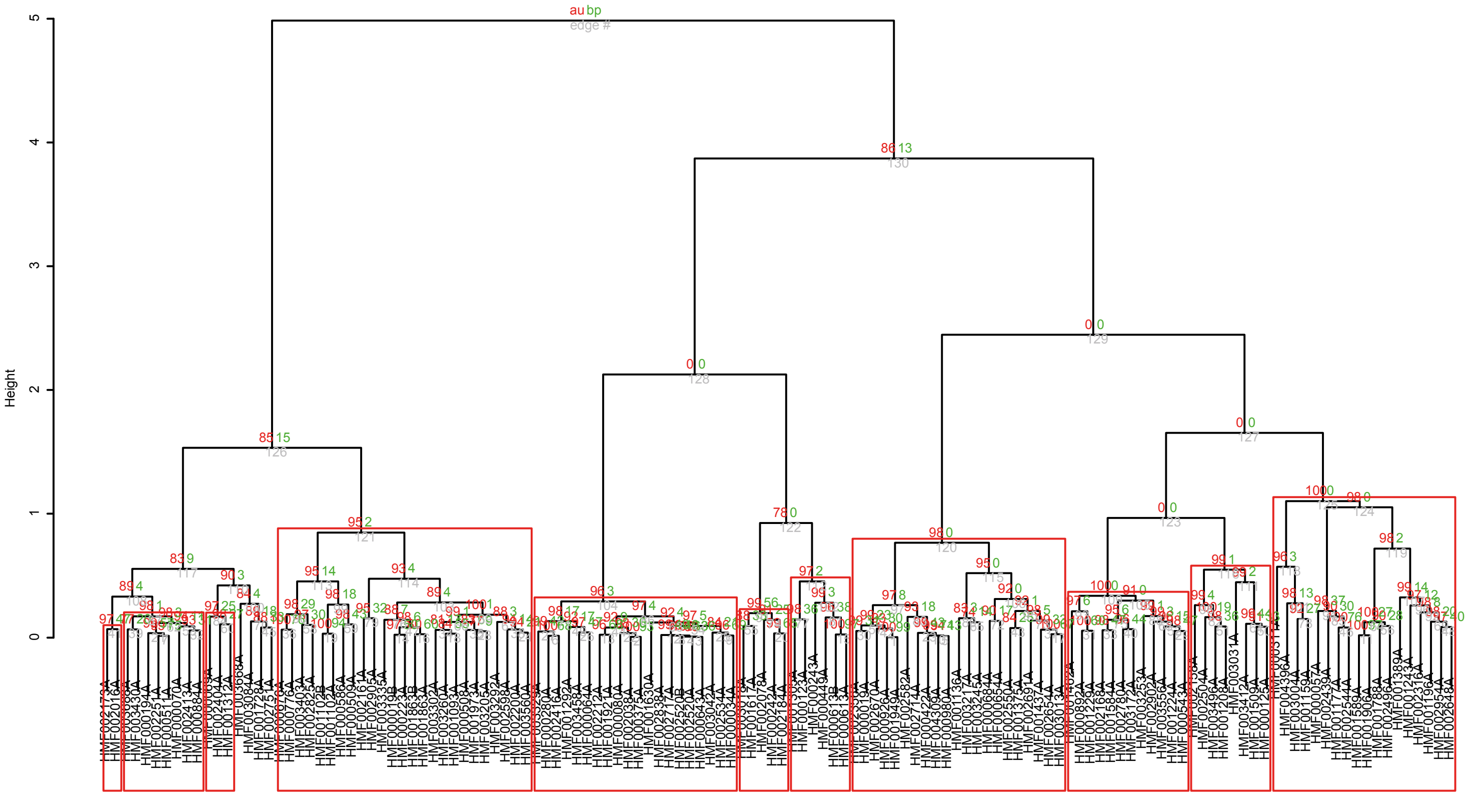

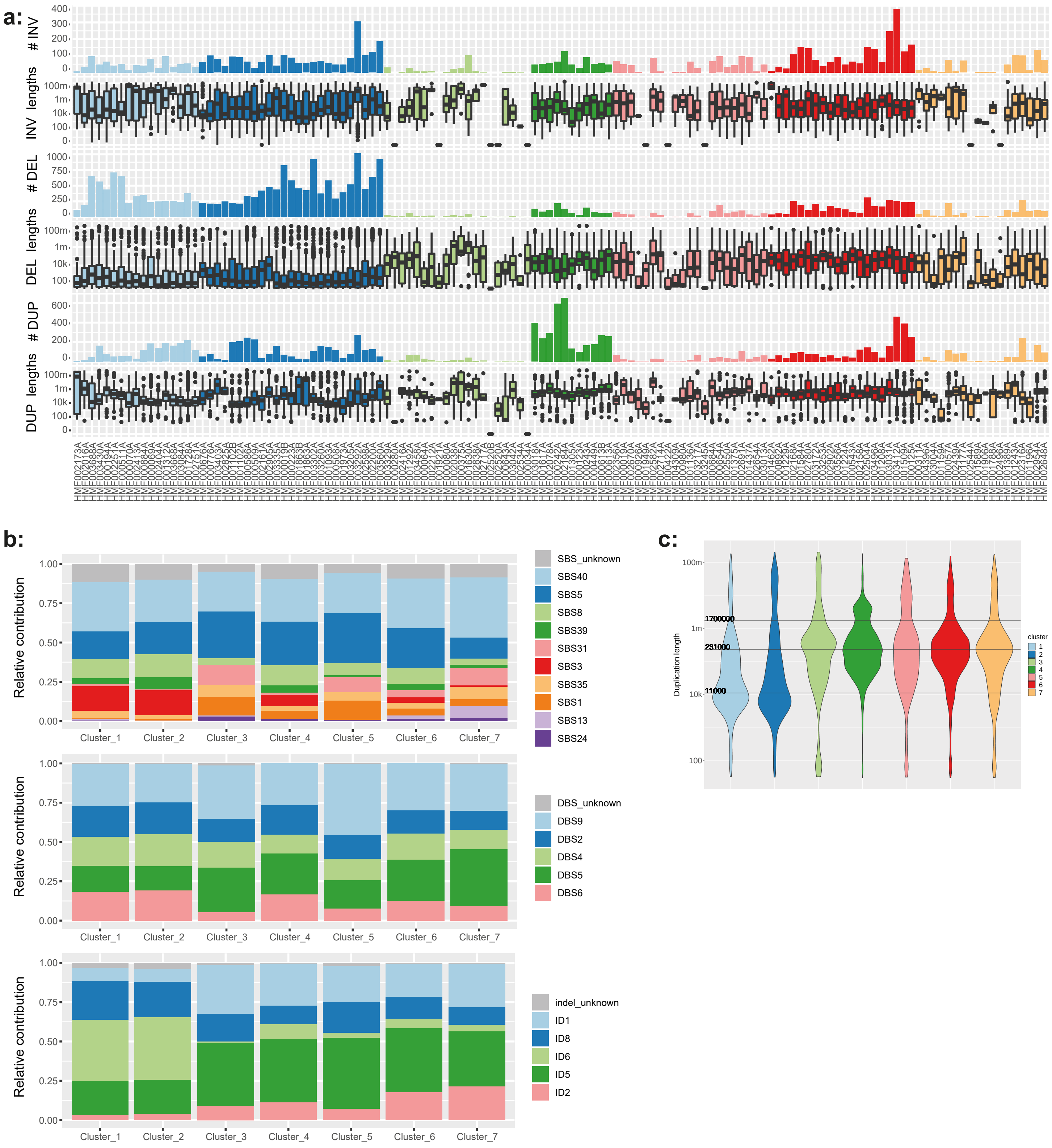

Figure S9. Distribution of SVs and mutational signatures per cluster. a. Number and length of inversions (INV), deletions (DEL) and duplications (DUP) per sample, grouped by cluster and presented in the order of the cluster plot (Fig. 4). b. Average relative contribution per cluster to each of the eight SBS, five DBS and five indel COSMIC signatures. Unknown indicates the fraction of mutations not explained by the named signatures. c. Duplication length per cluster.

a:

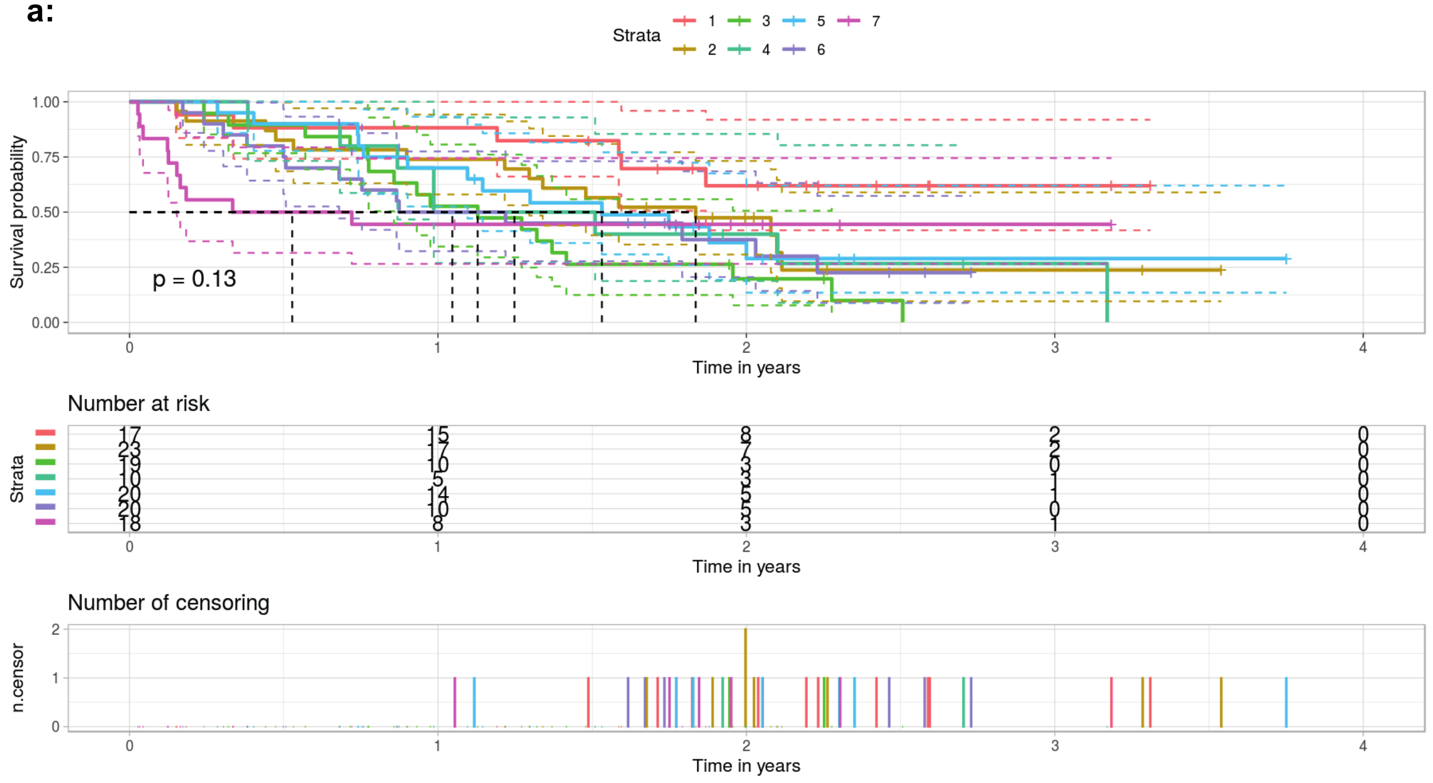

b:

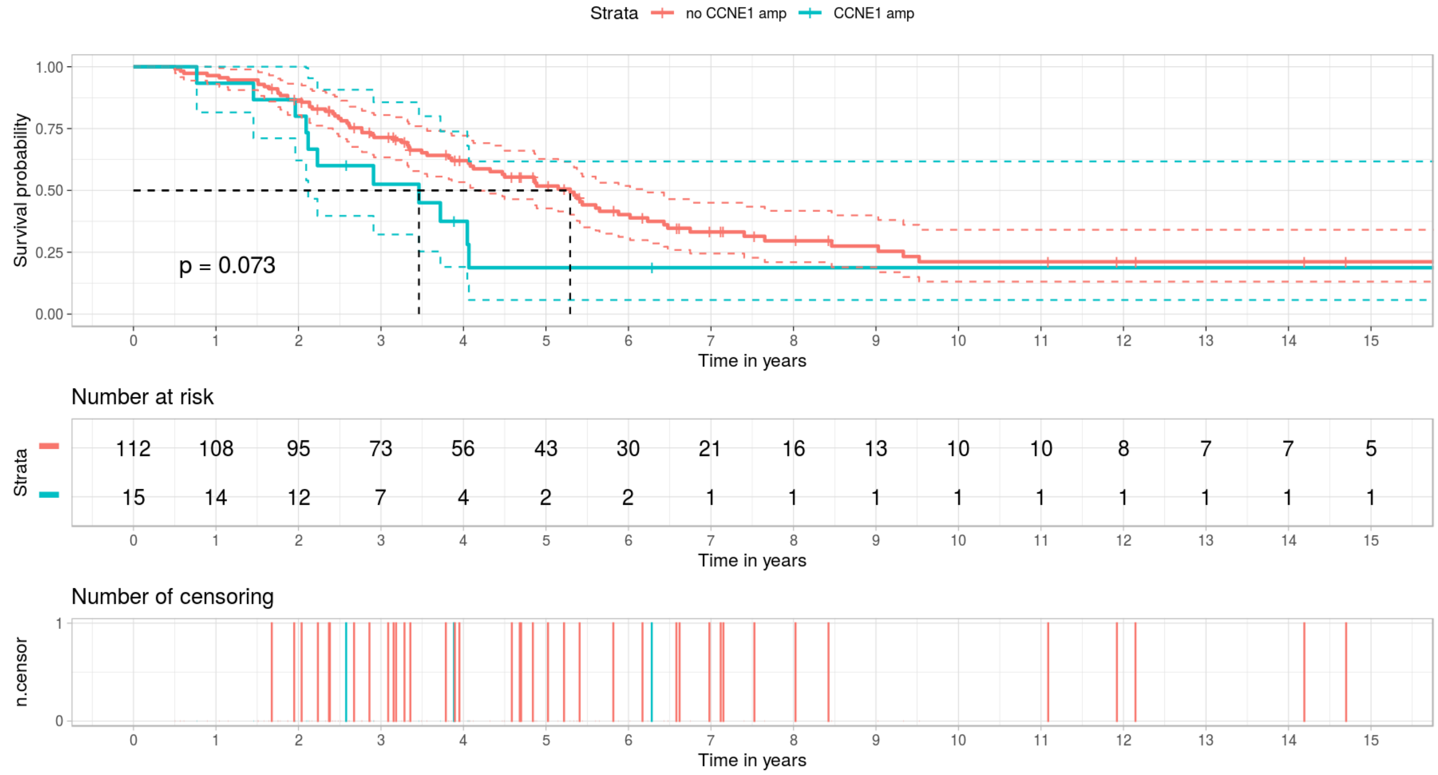

Figure S10: Survival probability per cluster (a:) and depending on CCNE1 amplification (b:).
Kaplan Meier plot indicating the survival probability (strata) in years from biopsy. Patients were censored at last follow-up, as indicated in the bottom of the plot. The colored dotted lines indicate the 95% confidence interval.
a: Survival per cluster **b:** Survival across cohort in presence of CCNE1 amplification vs. absence of CCNE1 amplification.

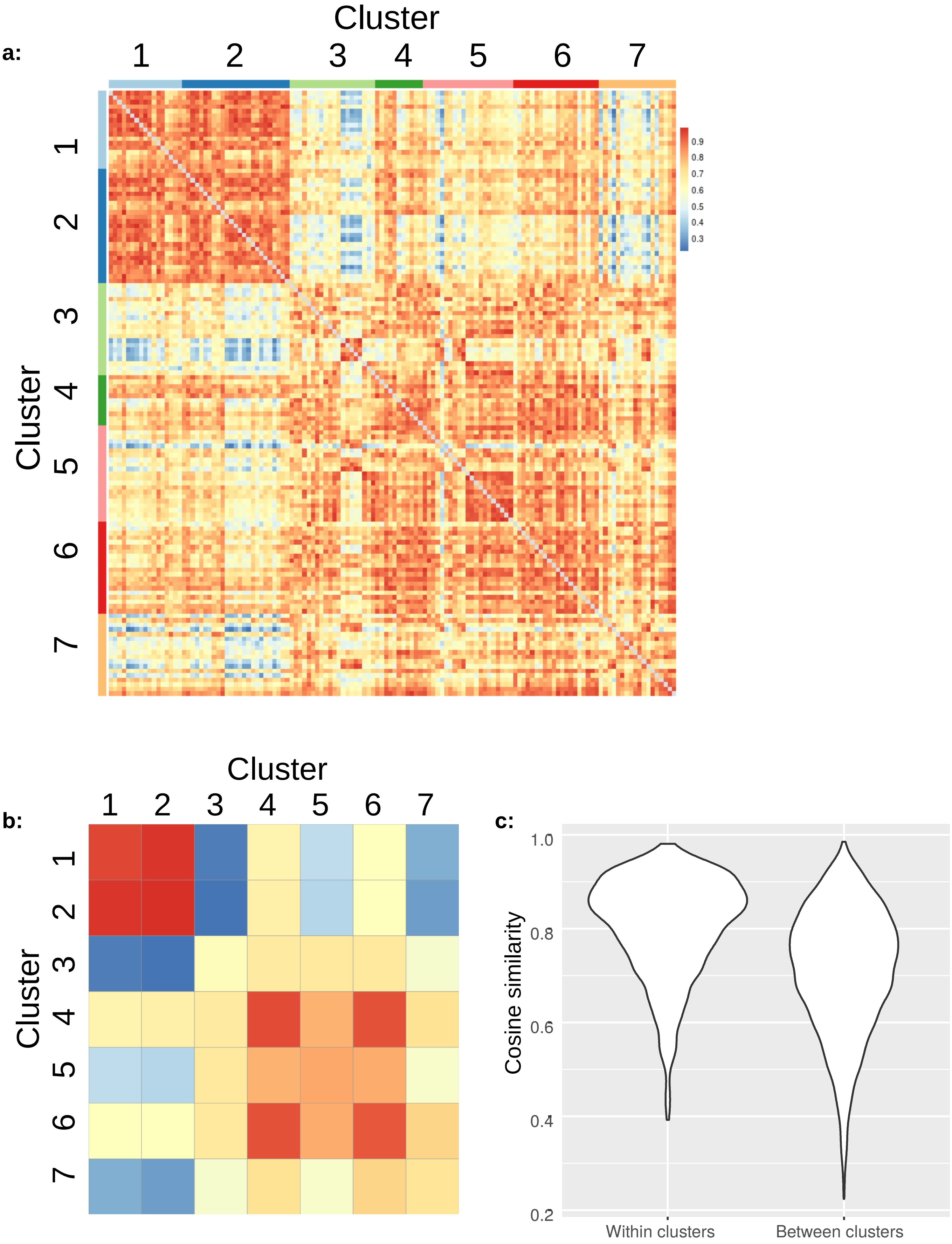

Figure S11: Cosine similarity of mutational signatures (SNV, DBS, INDEL combined) of samples within and in between clusters. a: Signature cosine similarity of all samples vs. each other, **b:** Average cosine similarity for all clusters vs. each other, **c:** similarity distribution of samples within and between clusters.

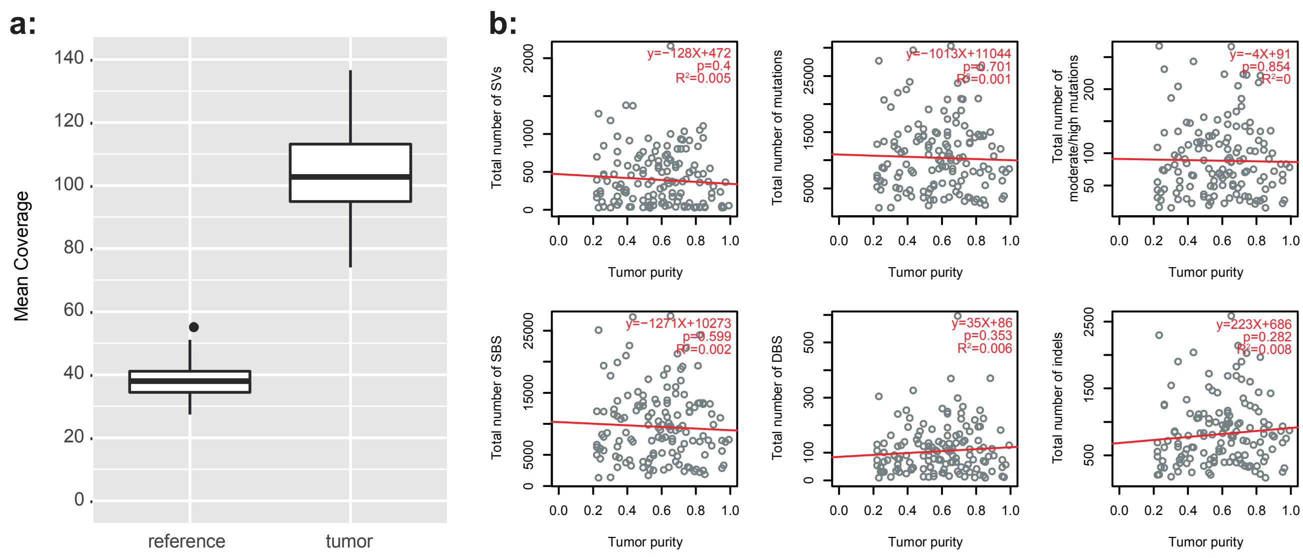

Figure S12. WGS quality control. a: Mean coverage of WGS for reference and tumor samples. **b:** Scatterplots, tumor purity versus number of SVs, mutations, moderate/high impact mutations, SBS, DBS and indels. Tumor purity (based on WGS, determined by PURPLE) did not influence the number of called variants ($p>0.05$).

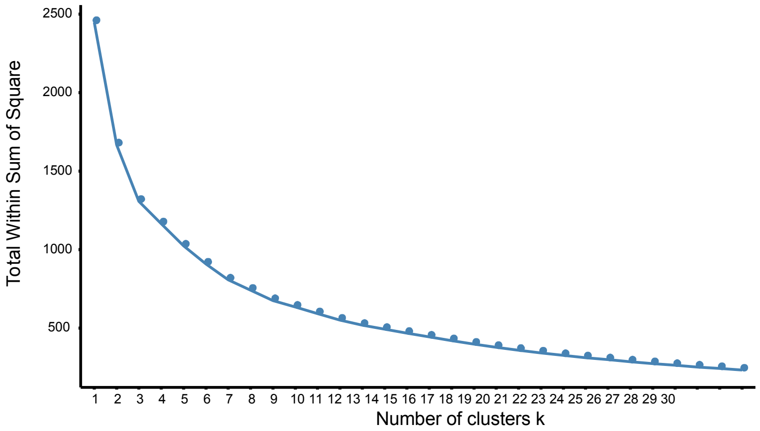

Figure S13: Elbow plot for the hierarchical clustering (Fig. 4). No clear bend was observed.
